# The role of early blood purification for miliary tuberculosis presenting as acute respiratory distress syndrome in pregnancy: A case report

**DOI:** 10.1097/MD.0000000000033523

**Published:** 2023-04-21

**Authors:** Anggraini Permata Sari, Muhammad Azhari Taufik, Krishna Wibisana, Widyastuti Sarkoen

**Affiliations:** a Department of Internal Medicine, Fatmawati Central General Hospital South Jakarta, DKI Jakarta, Indonesia; b Intensivist, Anesthesiology Department, Fatmawati Central General Hospital South Jakarta, DKI Jakarta, Indonesia; c Department of Obstetrics and Gynaecology, Fatmawati Central General Hospital South Jakarta, DKI Jakarta, Indonesia.

**Keywords:** acute respiratory distress syndrome, blood purification, miliary TB, pregnancy

## Abstract

Acute respiratory distress syndrome (ARDS) in miliary tuberculosis (TB) remains rare, especially in pregnant women. The role of blood purification is potential in managing ARDS due to miliary TB.

**Patient concerns:**

A 36-year-old woman presenting with difficulty breathing 6 hours before admission. She never had any constitutional symptoms due to TB.

**Diagnoses:**

ARDS in TB was diagnosed based on the deterioration of PaO_2_/FiO_2_, increased acute phase reactants, positive gene-Xpert, and typical chest x-ray of miliary TB.

**Interventions:**

A C-section was performed and followed by continuous venovenous hemofiltration to tackle her inflammatory condition. antituberculosis drugs were given after the transaminases showed declining trends.

**Outcomes:**

No major complications associated with continuous venovenous hemofiltration occurred. After 14 days of hospitalization, the patient’s clinical condition improved and was finally discharged.

**Lessons:**

This case underscores the potential role of blood purification in ARDS due to miliary TB in pregnancy.

## 1. Introduction

Miliary tuberculosis (TB) is a communicable disease that is a major cause of ill health and one of the leading causes of death worldwide. Until the coronavirus disease-19 pandemic, TB was the leading cause of death from a single infectious agent, ranking above human immunodeficiency virus (HIV)/acquired immunodeficiency syndrome.^[1]^ The most obvious impact on TB of disruptions caused by the coronavirus disease-19 pandemic is a large global drop in the number of people newly diagnosed with TB and reported in 2020, compared with 2019. Following large increases between 2017 and 2019, there was a fall of 18% between 2019 and 2020, from 7.1 million to 5.8 million.^[2]^

TB is caused by the bacillus *Mycobacterium tuberculosis*, which is spread when people who are sick with TB expel bacteria into the air (e.g., by coughing). Miliary TB is a potentially fatal form of TB. The prognosis of miliary tuberculosis per se has clearly improved after the introduction of effective antituberculosis drugs. But when acute respiratory distress syndrome (ARDS) develops after miliary tuberculosis, the fatality rate is far higher than other causes. ARDS in miliary TB remains rare, especially in pregnant women, and may develop due to reconstitution of immune system in pregnancy.^[3]^ Early initiation of blood purification in this patient was associated with improvement of oxygenation and inflammatory panels.

## 2. Case report

A 36-year-old Asian female, who works as a nurse, gravida 4 abortion 2 para 1 at 33 weeks, was admitted to our institution as she had difficulty breathing 6 hours before admission. There were no typical constitutional symptoms for TB such as weight loss, a decrease in appetite, fever, or cough. This pregnancy was planned by insemination, and all routine investigations including HIV screening were remarkable.

On admission, she was febrile, tachycardic, and tachypneic with an oxygen saturation of 88%. The patient was subsequently put on a high-flow nasal cannula (Flow 60 liter per minute, oxygen fraction 90%) due to respiratory deterioration. Arterial blood gas analysis revealed a PH of 7.47, pCO_2_ of 56.6, pO_2_ of 56.4, and SaO_2_ of 42.9%, and her PaO_2_/FiO_2_ ratio was 140, fulfilling the criteria of ARDS.

Auscultation of the lungs was significant for bilateral crackles. Her blood work was significant for normochromic normocytic anemia (9.5 g/dL), high D-dimer (3454 mg/mL), white count of 9900/µliter with 80% neutrophils, a lactate level of 2.6 millimoles per liter, C-reactive protein 28.04 mg/dL, and increase of transaminases more than 4 times upper limit normal, vitamin D-25OH was normal (38 ng/mL) (Table 1). Her chest X-ray revealed a diffuse interstitial alveolar pattern with asymmetrical consolidation with air bronchogram, most seen in the right lower lobe. Gene-Xpert and *M tuberculosis* cultures were performed, and the results were positive.

**Table 1:** Laboratory findings on admission.

LED	
Hemoglobin	10
Hematocrit	30
Leukocyte	9800
Thrombocyte	400,000
Neutrophil-lymphocyte ratio	5, 7
PT	13, 1
aPTT	30, 8
Aspartate transferase	133
Alanine transferase	139
Albumin	3, 29
D-dimer	3231
Fibrinogen	771
Ureum	14
Creatinine	0, 37
Natrium	132
Potassium	4
Chloride	96
Calcium ion	1,07
Magnesium	1,7
C-reactive protein	10, 08
Procalcitonin	1,37
Laktat	1.9
Blood glucose	81
HbA1C	5, 7
Total bilirubin	0, 62
Direct bilirubin	0, 58
Indirect bilirubin	0, 04
Uric acid	5, 1
Lactate dehydrogenase	351
pH	7, 47
pCO_2_	28, 3
pO_2_	84, 8
Base excess	−3, 4
HCO_3_	20, 6
SaO_2_	95, 7
Acid fast bacilli sputum	Negative
Gene-xpert MTB	Positive

Initial treatment for this patient includes broad-spectrum antibiotics (meropenem), and steroids for fetus lung maturity. antituberculosis drugs were postponed because of the abnormalities in transaminases. On the second day, the transaminases decreased after stronger neo-minophagen C was injected, and the antituberculosis (Rifampicin 600 mg INH 400 mg Ethambutol 600 mg) was started. Dexamethasone twice daily 6 mg was injected for fetal lung maturation. We planned to deliver the baby the next day.

A C-section was performed on day 3, after the operation, the cytokine panel was checked, and the results were come out to be all high cytokine levels (Table 2). The patient was put on ventilator support (VC 300 mL, Fraction 70%, PEEP 6 cm H_2_O) and continuous venovenous hemofiltration (CVVH) was performed with a modified AN69ST hemofilter for 24 hours. Pyrazinamide 1000 mg was started after CVVH was finished.

**Table 2:** A follow-up blood test after performing CVVH showed a decline in the inflammatory parameters and cytokine panels.

	Normal range	One day before CVVH	4 Days post CVVH
Interleukin-6 (pg/mL)	0.7–12.5	14.42	7.54
Interleukin-10 (pg/mL)	<5	12.66	3.51
TNF α (pg/mL)	<8.8	76.16	20.4
IFN γ (pg/mL)	<5.0	355.29	15.1

CVVH = continuous venovenous hemofiltration.

**Figure 1.**
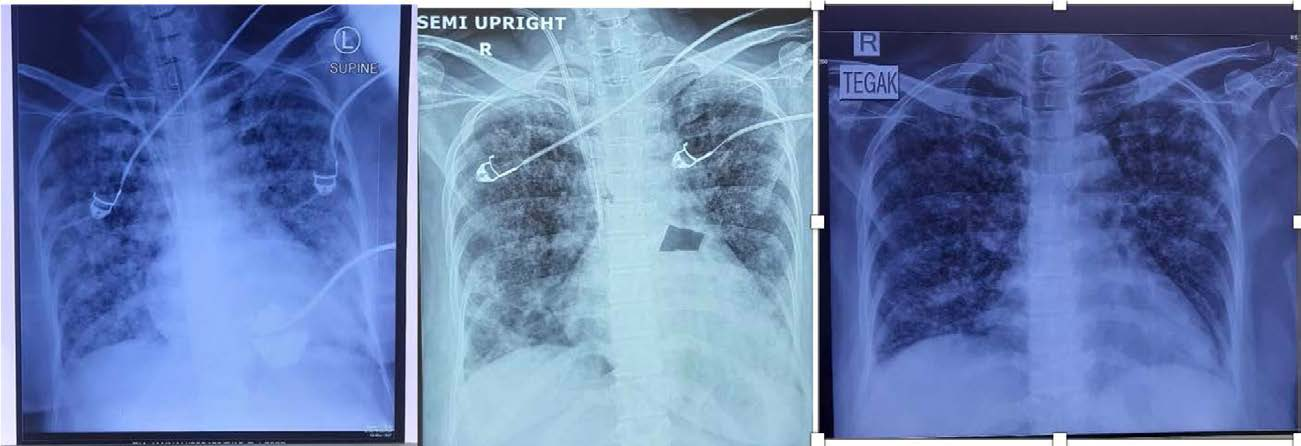
Serial chest X-rays.

After CVVH finished, the patient successfully passed the spontaneous breathing trial and get extubated the next day. Her clinical symptoms and chest X-rays also showed improvement within days (Fig. 1). After 14 days of hospitalization, the patient can be discharged. Her baby is also in good condition without any respiratory problems.

## 3. Discussion

Globally in 2020, about 84% of TB deaths among HIV-negative people and 85% of the combined total of TB deaths in HIV-negative and HIV-positive people occurred in the WHO African and South-East Asia regions.^[2]^

Miliary TB is a potentially fatal form of the disseminated disease due to the hematogenous spread of tubercle bacilli to the lungs, and other organs,^[4,5]^ It also features as an unusual cause of ARDS, remains rare, occurring in <1% to 2% of cases, and is usually associated with risk factors including HIV/acquired immunodeficiency syndrome, an ever-increasing list of causes of immunosuppression, such as the use of biologicals and immunosuppressive drugs for the treatment of various medical disorders, increasing occurrence of organ transplantation, chronic hemodialysis program, among others.^[6]^

The prognosis of miliary tuberculosis is quite promising, however, the situation is quite different when ARDS develops after miliary tuberculosis. According to the limited number of articles reporting the outcome of ARDS caused by miliary tuberculosis, the fatality rate ranges from 33% to as high as 100%,^[1,5]^ which is far higher than for ARDS from other causes. Such a poor prognosis demands significant improvement in the management of this critical condition.^[7]^

Although ARDS may develop anytime during the course of miliary TB, it is usually seen at the time of initial presentation. ARDS may develop as a component of the multiorgan dysfunction syndrome due to TB or as a manifestation of immune reconstitution inflammatory syndrome.^[6]^

TB in pregnancy itself poses a substantial risk of morbidity to both the pregnant woman and the fetus if not diagnosed and treated promptly. In the first and second semesters, the immune system of a pregnant patient was decreased due to the implantation phase. But during late pregnancy, the fetus has completed its development and the mother needs to deliver the baby, this is achieved through renewed inflammation.^[3]^ Since this patient on the late phase of pregnancy, the renewal inflammation phase was likely has the same effect as immune reconstitution inflammatory syndrome, and it provoked this patient to have ARDS.

Another thing, the neutrophil-lymphocyte ratio (NLR) in this patient was considered in a high ratio (5.7). NLR in pregnant patients is usually elevated in the second trimester and goes to a normal ratio in the third semester.^[8]^ NLR > 5 in combination with band neutrophils can be the predictor of inflammatory progression and indicate unfavorable prognoses in the patient.^[9]^

Early initiation of blood purification is associated with favorable clinical outcomes in ARDS patients, which might be due to reduced inflammatory panels.^[5]^ This patient was thought to have elevated cytokines due to ARDS plus postoperative C-section, and this was the reason why the timing of blood purification was started after the patient had a C-section.

After the patient had blood purification and administration of the antituberculosis drug, the clinical condition (defined by decreased oxygen requirement, improved oxygenation, and the amelioration of chest X-ray findings) was improved after 24 hours. This case is an example of successful treatment of ARDS due to miliary TB in a pregnant patient with early blood purification in combination with an antituberculosis strategy.

## 4. Patient perspective

When I first found out that my shortness of breath was most likely due to miliary TB, the severe 1, I was shocked because I knew that this could be my end of life. I had no history of contact with TB patients, especially the miliary type. I am a nurse; I knew this could affect my precious baby. we have been waiting so long to have this baby, after several failures years ago.

The doctors moved me into ICU, they talked to me and my husband about their plans. They told us about the complications of the procedures, the worst 1, is my death. When they put me into a high-flow respirator, I was so scared, but I trust my doctors. The next day, they did a C-section and after that, I remembered nothing.

I woke up and realized that my belly was already flat and I was put on a ventilator. I was confused, about how many days I already skipped and what happened to my baby. My fellow nurses told me that the doctors has already done the C-section and run CVVH, and right now I was saved and fully alive. I had very great support after the aggressive therapy, and after 14 days I can be discharged from the hospital. I was so proud of my doctors and my fellow nurses; they worked together as a tremendous team to save my life.

## Author contributions

**Conceptualization:** Anggraini Permata Sari, Muhammad Azhari Taufik, Widyastuti Sarkoen.

**Data curation:** Anggraini Permata Sari.

**Investigation:** Muhammad Azhari Taufik, Krishna Adi Wibisana, Widyastuti Sarkoen.

**Supervision:** Widyastuti Sarkoen.

**Validation:** Anggraini Permata Sari, Krishna Adi Wibisana.

**Writing – original draft:** Anggraini Permata Sari, Muhammad Azhari Taufik.

**Writing – review & editing:** Anggraini Permata Sari.

## References

[1] Mert A Arslan F Kuyucu T. Miliary tuberculosis: epidemiological and clinical analysis of large-case series from moderate to low tuberculosis endemic country. Medicine (Baltim). 2017;96:e5875. 10.1097/MD.0000000000005875 PMC5293426 28151863

[2] Impact of the COVID-19 Pandemic on TB Detection and Mortality in 2020. Geneva: World Health Organization; 2021. Available at: https://www.who.int/publications/m/item/impact-of-the-covid-19-pandemic-on-tb-detection-andmortality-in-2020

[3] Mor G Cardenas I. The immune system in pregnancy: a unique complexity. Am J Reprod Immunol. 2010;63:425–33. 20367629 10.1111/j.1600-0897.2010.00836.x PMC3025805

[4] Mohan A Sharma SK Pande JN. Acute respiratory distress syndrome in miliary tuberculosis: a 12-year experience. Indian J Chest Dis Allied Sci. 1996;38:147–52. 8987289

[5] Han F Sun R Ni Y. Early initiation of continuous renal replacement therapy improves clinical outcomes in patients with acute respiratory distress syndrome. Am J Med Sci. 2015;349:199–205. 25494217 10.1097/MAJ.0000000000000379

[6] Sharma SK Mohan A Sharma A. Miliary tuberculosis: new insights into an old disease. Lancet Infect Dis. 2005;5:415–30. 15978528 10.1016/S1473-3099(05)70163-8

[7] Carmen T Bte Zailan L Singh R. Miliary tuberculosis presenting as pyrexia of unknown origin in pregnancy. J Clin Gynecol Obstet. 2019;8:114–7.

[8] Klement AH Hadi E Asali A. Neutrophils to lymphocytes ratio and platelets to lymphocytes ratio in pregnancy: a population study. PLoS One. 2018;13:e0196706. 29787560 10.1371/journal.pone.0196706 PMC5963784

[9] Huang Z Fu Z Huang W. Prognostic value of neutrophil-to-lymphocyte ratio in sepsis: a meta analysis. Am J Emerg Med. 2020;38:641–7. 31785981 10.1016/j.ajem.2019.10.023

